# Targeted intestinal delivery of incretin secretagogues—towards new diabetes and obesity therapies

**DOI:** 10.1016/j.peptides.2017.11.008

**Published:** 2018-02

**Authors:** Fiona M. Gribble, Claire L. Meek, Frank Reimann

**Affiliations:** a Institute of Metabolic Science, Metabolic Research Laboratories, University of Cambridge, Addenbrooke’s Hospital, Box 289, Hills Road, Cambridge, CB2 0QQ, United Kingdom; b Department of Clinical Biochemistry, Addenbrooke’s Hospital, Hills Road, Cambridge, CB2 0QQ, United Kingdom

**Keywords:** Enteroendocrine hormones, peptideYY (PYY), Glucagon-like peptide-1 (GLP-1), Diabetes, Obesity, Capsules, Pellets, Infusion

## Abstract

A new strategy under development for the treatment of type 2 diabetes and obesity is to mimic some of the effects of bariatric surgery by delivering food-related stimuli to the distal gastrointestinal tract where they should enhance the release of gut hormones such as glucagon-like peptide-1 (GLP-1) and peptideYY (PYY). Methods include inhibition of food digestion and absorption in the upper GI tract, or oral delivery of stimuli in capsules or pelleted form to protect them against gastric degradation. A variety of agents have been tested in humans using capsules, microcapsules or pellets, delivering nutrients, bile acids, fatty acids and bitter compounds. This review examines the outcomes of these different approaches and supporting evidence from intestinal perfusion studies.

## Highlights

• Bariatric surgery dramatically elevates plasma glucagon-like peptide-1 (GLP-1) and peptideYY (PYY) levels.
• Targeted delivery of nutrients to the distal intestine also raises plasma GLP-1 and PYY levels.
• Encapsulated and pelleted GLP-1 and PYY secretagogues for distal intestinal delivery had so far only limited success.
• Future attempts should focus on highly potent agonists and take a possible need for absorption into account.

## 1 Introduction

Type 2 diabetes mellitus is an increasing cause of morbidity and mortality across the world and is strongly associated with obesity. Despite recent therapeutic advances, there remains an urgent need to identify new ways of treating diabetes and obesity. A major focus of new therapeutics is the enteroendocrine system, which produces a wide range of peptide hormones including glucagon-like peptide 1 (GLP-1), glucose-dependent insulinotropic polypeptide (GIP), cholecystokinin (CCK) and peptideYY (PYY). These act as hormonal signals linking food absorption to physiological responses such as insulin secretion and satiety. GLP-1 mimetics and inhibitors of GLP-1 degradation (DPP4 inhibitors) are in widespread use for the treatment of type 2 diabetes, and GLP-1 mimetics are also licensed for the treatment of obesity [1]. Bariatric surgery is an even more effective therapy for diabetes, and is believed to act, at least in part, by mobilising endogenous stores of gut hormones such as GLP-1 and PYY by shifting food absorption from the upper to the lower small intestine, where these hormones are found in higher abundance [2]. Reproducing the hormonal changes observed after gastric bypass surgery using pharmacological or dietary manipulations is regarded as a potential new strategy for treating diabetes and obesity. One proposed method is to target the delivery of nutritional and other stimuli to enteroendocrine cells in the lower gut by approaches such as delayed digestion/absorption, stimulus encapsulation, or harnessing of microbial metabolism to produce locally active secretagogues. This review explores recent progress in, and the potential future of, this approach.

## 2 Enteroendocrine cell location and function

Enteroendocrine cells are located in the epithelium of the gastrointestinal tract from the stomach to the rectum. They mostly span the epithelial layer, having surfaces facing both the gut lumen and the basolateral space, and respond to a variety of nutritionally-related stimuli including fats, carbohydrates, proteins and bile acids [3]. The detection of ingested foods occurs downstream of food digestion, requiring the formation of small molecular species such as glucose, free fatty acids, monoacylglycerols, amino acids, di/tripeptides and bile acids. These are detected by a variety of G-protein coupled receptors and nutrient transporters, which are highly, and in many cases specifically, expressed on enteroendocrine cell populations [3].

In a number of cases it has been found that stimulus detection by enteroendocrine cells requires local ligand absorption. Detection of glucose, for example, is directly linked to its influx across the apical membrane of enteroendocrine cells by sodium coupled glucose transporters (SGLT1), which causes depolarisation of the cell membrane and voltage gated calcium entry [4]. Detection of long chain fatty acids and bile acids also seem to occur downstream of their local absorption, but in these cases the requirement is to bring ligands into contact with their target enteroendocrine cell receptors (FFA1 and GPBAR1) apparently located on the basolateral membrane [5], [6]. Thus, in the upper small intestine, chylomicron formation is a key step linking ingested fat to the secretion of GLP-1, CCK and GIP [7], [8]; and in the distal ileum, bile acid-triggered GLP-1 secretion occurs downstream of conjugated bile acid absorption by sodium coupled bile acid transporters [5]. The linkage of enteroendocrine cell activation to local nutrient absorption has the consequence that these ligands do not trigger gut hormone release if they only make transient contact with the apical surfaces of enteroendocrine cells as they pass by in the intestinal lumen, but only act if they are being actively absorbed in the local vicinity.

Many features of enteroendocrine cells, including their morphology and signalling pathways, are shared along the length of the gut, but there are marked regional differences in the hormones they produce. GIP and CCK, for example, are predominantly produced in the proximal small intestine, whereas GLP-1 and PYY are found at higher levels in the lower small intestine and colon [9]. Although proximal to distal signalling loops that recruit distal GLP-1 in response to proximal nutrient detection have been proposed [10], [11], when nutrients are absorbed in the upper gut, they trigger a very different profile of gut hormones compared with when nutrient absorption is shifted to more distal regions of the gut. During normal physiology, the upper small intestine is the major site of nutrient digestion and absorption and the gut employs a battery of neurohormonal feedback pathways to limit the quantity of potentially absorbable food reaching the ileum and colon and the consequent risk of losing nutrients in the faeces. Pharmacological and surgical interventions, however, can overcome these systems and deliver large loads of nutrients into the distal gut, resulting in major shifts in post-prandial gut hormone profiles.

## 3 Gut hormone changes after bariatric surgery

Surgeons have developed a variety of bariatric procedures, including gastric banding, Roux-en-Y gastric bypass (RYGB) and sleeve gastrectomy, that are each effective therapies for obesity but have different effectiveness on type 2 diabetes [2]. Many studies have demonstrated that RYGB and sleeve gastrectomy, but not gastric banding, have beneficial effects on glucose metabolism and diabetes beyond those predicted by weight loss alone, but the underlying explanations remain disputed and are likely multifactorial. RYGB and sleeve gastrectomy have a number of physiological consequences that might contribute to weight loss and enhanced insulin secretion, including changes in the enteroendocrine axis [12], [13], increased plasma bile acids [14] and calorie restriction [15]. Both procedures result in an increased rate of nutrient delivery to the distal gut, because the stomach is either bypassed or cut down to form a tube with limited storage capacity. RYGB and sleeve gastrectomy have been associated with dramatically elevated post-prandial plasma concentrations of GLP-1 and PYY [2], and are likely accompanied by a range of other gut hormone changes consequent upon the predominant direct stimulation of distal as opposed to proximal enteroendocrine cells.

The following sections outline different approaches that have been taken to mimic the effects of gastric bypass surgery by delivering stimuli to endocrine cells in the distal gut. In this review, we take the position that measurable elevations in plasma hormone concentrations will be a good marker of an effective approach to increase secretion from distal enteroendocrine cells, although subtle, locally-restricted increases in distal gut hormone levels might have their own beneficial effects, given that GLP-1 receptors are found in the direct vicinity of the secreting cells [16].

## 4 Experimental intestinal infusions for targeted stimulus delivery

To investigate how gut hormone release is influenced by the position at which nutrients are delivered, a number of investigators have used controlled nutrient infusions into the stomach, duodenum and jejunum of humans. Gastric infusions enable the bypassing of the oropharynx, and therefore the taste and olfactory cues associated with eating. Although there is little evidence to suggest that gut hormone responses are triggered by the cephalic phase of eating, these studies do enable a defined rate of nutrient delivery that is difficult to achieve by oral ingestion. Gastric infusions cannot, however, provide a guaranteed rate of nutrient delivery into the small intestine because the pylorus acts downstream of the infusion, and closely regulates gastric emptying.

Duodenal infusions have been studied to investigate the dependence of gut hormone secretion on the nutrient stimulus and its rate of delivery. These studies have established that low dose infusions of carbohydrates, fats and proteins are each able to stimulate secretion of hormones produced by the duodenum (GIP, CCK), but that higher infusion rates are required to trigger a robust elevation of plasma GLP-1 or PYY [17], [18], [19]. The proposed explanation is that higher infusion rates exceed the maximal absorption capacity of the upper gut and therefore result in nutrient delivery more distally where there is a higher density of cells producing GLP-1 and PYY.

A few studies have placed cannulae more distally in the gut and demonstrated robust elevation of GLP-1 and PYY secretion in response to glucose [20], [21]. In interpreting results from infusion studies performed lower down the intestine, it is important to remember that only the digestion products of macromolecules are effective gut hormone secretagogues, and that some molecules are dependent on sodium for their absorption. Delivery of macromolecular polymers that require pancreatic enzymes for their digestion is therefore unlikely to trigger a large secretory response, and infusion of glucose will have limited effect unless there is an adequate supply of sodium ions from the infusion itself, bile or local secretion.

In situ intestinal perfusion studies have also been carried out using animal models, such as the pig, rat or mouse. In these studies, the intestinal arterial supply is isolated for vascular perfusion and the portal vein cannulated for venous collection. Additional cannulae in the gut lumen enable luminal perfusion of candidate stimuli. These studies enable stimulus delivery to a defined segment of the gut, and can target intestinal regions not readily accessible in man, such as the distal ileum and colon. They permit the co-application of candidate pharmacological inhibitors and agonists and therefore enable a closer interrogation of the pathways underlying nutrient detection. Comparisons between the effectiveness of different stimuli delivered either through the lumen or vasculature have confirmed, for example, that luminal glucose only triggers gut hormone secretion in the presence of luminal sodium ions [22], that bile acids are more effective when delivered via the vasculature than from the lumen [5], and that the long chain free fatty acid receptor FFA1 is more accessible from the vascular than the luminal direction [6].

## 5 Pharmacological agents causing increased delivery of stimulant to the distal gut

There are a number of existing therapies that increase GLP-1 secretion in humans likely because they increase nutrient delivery to the distal gut. A variety of mechanisms have been implicated, including reduced nutrient absorption in the proximal intestine and inhibition of nutrient digestion.

### 5.1 Alpha glucosidase inhibitors

Acarbose, miglitol and voglibose are alpha glucosidase inhibitors that slow starch and sucrose digestion in the proximal gut, leading to increased delivery of undigested carbohydrate to the distal intestine. Plasma GLP-1 levels were higher in healthy volunteers [23] and patients with T2DM treated with acarbose following sucrose ingestion [24] but not after a mixed meal [25], whereas concentrations of GIP were lowered by acarbose in patients with T2DM [24], [25]. In a group of Japanese patients with T2DM, administration of voglibose or miglitol for 12 weeks increased GLP-1 and reduced GIP, insulin and glucose concentrations after a meal test [26]. Similar results were seen in a 24-week study of patients with insulin-treated type 1 diabetes (T1DM) [27], in which the miglitol treated group had a small increase in GLP-1, reduced GIP, needed slightly less exogenous insulin and had fewer pre-prandial hypoglycaemic events. Despite these potential benefits, the magnitude of the effect on GLP-1 levels has been relatively small, perhaps because doses of the alpha glucosidase inhibitors that are sufficient to reduce carbohydrate digestion in the upper gut will similarly reduce digestion in the distal gut, thereby also restricting the local release of glucose from complex carbohydrates in the vicinity of distal enteroendocrine cells.

### 5.2 SGLT1/2 inhibitors

An alternative method to reduce glucose absorption in the proximal gut is to inhibit the intestinal brush border glucose transporter, SGLT1. Inhibitors of the renal glucose transporter SGLT2 are now widely used in clinical practice, and mostly have limited efficacy on SGLT1. Canagliflozin and sotagliflozin, however, are mixed SGLT1/2 inhibitors that reduce intestinal as well as renal glucose absorption [28]. Both drugs have been associated with elevated post-prandial GLP-1 and PYY levels in human subjects [29], [30]. The proposed mechanism is by shunting glucose to the distal gut, where there is a higher density of GLP-1 and PYY producing enteroendocrine cells. There is some debate about how glucose is sensed once it reaches the distal gut, as enteroendocrine cells might arguably not be able to use SGLT1 as a glucose sensor in the presence of an SGLT1 inhibitor. However, it is unlikely that SGLT1 inhibition is complete in patients on these drugs because the rate of glucose appearance from the intestine was only transiently impaired after canagliflozin treatment [30], and glucose levels still rose post-prandially in the plasma of patients on sotagliflozin therapy [29]. Nevertheless, distally located enteroendocrine cells might additionally employ an alternative glucose sensor, as elevated post-prandial GLP-1 levels were also observed in mice completely lacking SLGT1 [31]. Such a pathway could detect products of anaerobic fermentations, such as short-chain fatty acids [32] or involve sweet taste receptors (Tas1R2/3), although a role of the latter in glucose triggered GLP-1 secretion has been controversial [3].

### 5.3 Orlistat

Orlistat is an inhibitor of pancreatic and intestinal lipases that prevents lipid digestion in the intestinal lumen and causes increased distal delivery of undigested fats. Depending on the extent of lipase inhibition, this might, or might not, trigger increased gut hormone secretion, because digestion to fatty acids and monoacylglycerols is itself a prerequisite for activation of enteroendocrine cells. Indeed, a single dose of orlistat was found to lower post-prandial GLP-1, CCK and PYY levels and satiety in healthy volunteers [33]. Smaller studies in obese non-diabetic and type 2 diabetic subjects, examined using a smaller test meal, found no change or a small increase in post-prandial GLP-1 following a single dose of orlistat [34], [35]. Overall, the results suggest that although orlistat delivers an increased fat load to the distal gut, this has only limited ability to stimulate GLP-1 secretion, probably because the persistent lipase inhibition causes reduced release of fatty acids and monoacylglycerols in the distal intestine. The importance of fat digestion for incretin secretion is supported by the observed elevations of postprandial GIP and GLP-1 levels after pancreatic enzyme supplementation in patients with chronic pancreatitis [36], and cystic fibrosis [37]. The ability of distally-released free fatty acids to stimulate GLP-1 and PYY secretion is also likely influenced by the efficiency of fat absorption and chylomicron biosynthesis in the distal compared with the proximal small intestine, because gut hormone release appears to require fatty acid stimuli to access FFA1 on the basolateral surface of enteroendocrine cells [6].

### 5.4 Bile acid sequestrants and inhibitors of bile acid uptake

Bile acids are a potent stimulus of GLP-1 secretion in vitro and in perfused intestine, but as discussed above must be absorbed across the epithelium to activate the bile acid receptor GPBAR1 (also known as TGR5) on enteroendocrine cells. In human studies, jejunal infusion of taurocholic acid (2 g) had no effect on plasma GLP-1 levels [38], but rectal administration of 0.4–11.8 g sodium taurocholate or 1.5–3.5 g taurocholic acid by enema increased both GLP-1 and PYY concentrations [39], [40]. Bile acid sequestrants also deliver increased loads of bile acids to the distal gut by sequestering them within the gut lumen. One line of thought has suggested that an increased bile acid load to the distal gut achieved by bile acid sequestration might similarly enhance GLP-1 secretion, but the recent finding that absorption is required for bile acids to access GPBAR1 on enteroendocrine cells has called this into question. Indeed a study in humans showed that gastric infusion of chenodoexycholic acid (1.25 g) increased plasma GLP-1 concentrations, but that this was abolished in the concomitant presence of the bile acid sequestrant colesevelam [41], indicating that bile acids complexed in the gut lumen with a sequestrant are unable to activate enteroendocrine cells. An alternative approach of inhibiting bile acid absorption in the terminal ileum would also be predicted to increase their delivery to colonic enteroendocrine cells. There is currently little data to support this idea in humans, but a study of patients with dyslipidaemia treated with the ileal bile acid transporter inhibitor elobixibat showed elevated peak post prandial GLP-1 levels, although otherwise normal plasma GLP-1 concentrations [42].

### 5.5 Metformin

Metformin is currently the first-line agent for the treatment of T2DM but its mode of action is imperfectly understood. A number of recent studies have suggested that its primary target is located in the gut. Metformin increases plasma GLP-1 concentrations and may also reduce DPP-IV activity in patients with T2DM [43], but there is little evidence to suggest the drug acts directly on enteroendocrine cells [44]. An alternative idea is that the effect of metformin on GLP-1 concentrations is indirect, acting for example through reduced intestinal absorption of glucose or bile acids [45]. Metformin-treated patients with type 2 diabetes exhibited elevated plasma GLP-1 levels and reduced glucose absorption from the gut lumen during a duodenal glucose infusion, with a significant correlation between the GLP-1 increment and the impairment of luminal glucose uptake [46]. In rats, metformin was also a potent inhibitor of ileal bile acid absorption [47], potentially explaining the altered plasma bile acid concentrations observed in metformin-treated patients [45]. These results suggest that increased glucose delivery to the lower small intestine or bile acid delivery to the colon could contribute to the enhancement of GLP-1 secretion in metformin treated patients.

## 6 Stimulus delivery to the distal gut by delayed release formulations

Rather than chemically preventing stimulus production or absorption in the proximal gut, an alternative method to deliver stimuli to the lower gut is by the use of formulations that only release their chemical components after a time delay, in response to a change in pH, or following bacterial fermentation. Using these approaches, a variety of candidate GLP-1 secretagogues have been delivered to the ileum and colon of humans, with variable success.

### 6.1 Free fatty acids

Short chain fatty acids (SCFA) stimulate GLP-1 secretion in vitro [32] and increase numbers of GLP-1 secreting L-cells [48], but the magnitude of their effect on hormone levels in humans remains controversial. Strategies that have been tested for distal delivery of SCFA include encapsulation and fermentable fibre (e.g. inulin, resistant starch). Although high fibre diets have been associated with elevated plasma GLP-1 and PYY levels [49], acute administration of SCFA capsules, inulin or resistant starch had minimal effects on GLP-1 concentrations. Overweight and lean subjects were tested with an acute dose of inulin (24 g) or resistant starch (28 g) together with an oral glucose tolerance test, and although plasma acetate, propionate and butyrate concentrations were substantially elevated by the treatments, particularly in the inulin-treated participants [50], there were no differences in GLP-1 or PYY concentrations between the groups [51]. In a study of sodium butyrate capsules, type 2 diabetic participants took six 100 mg capsules before and after each meal, six times a day for 45 days (7.2 g per day), with or without inulin, and exhibited small changes in blood glucose and waist hip ratio, but little change in post-prandial GLP-1 [52]. Taken together, these results suggest that acute SCFA release in the distal gut has little effect on secretion from enteroendocrine cells in humans, and that longer term delivery has only marginal effects on GLP-1 levels. High fibre diets might have alternative beneficial effects by enhancing delivery of other ingested nutrients to the distal intestine.

Gut hormones have also been measured in humans during intraduodenal infusion of the medium chain length 12-carbon fatty acid, lauric acid, or the long chain 18-carbon fatty acid, oleic acid. Lauric acid (2.7 g) or oleic acid (2.9 g), infused into the duodenum over a one hour time period, were strong stimuli of CCK and PYY secretion [53], likely acting via FFA1 [54]. Lauric acid was subsequently formulated as enteric-coated pellets and given to a group of type 2 diabetic patients, who each received pellets containing 2.4 g lauric acid (or placebo) before breakfast and before lunch on a single study day. Plasma GLP-1 but not GIP was increased, and plasma glucose was lowered in the lauric acid treated group [55]. The results suggest that lauric acid is an effective stimulus of enteroendocrine cells in humans. The lack of effect of pelleted lauric acid on GIP might reflect the release of active stimulant only in the more distal gut where GIP is less abundant.

### 6.2 Amino acids

Glutamine was identified as a stimulus of GLP-1 secretion in vitro [56], and was subsequently found to trigger GLP-1 secretion in healthy volunteers [57] and to improve glucose tolerance in patients with type 2 diabetes when administered orally [58]. Studies to identify the underlying receptor in enteroendocrine cells have identified sodium coupled amino acid transporters and the calcium sensing receptor as potential candidates [59], [60]. High oral doses (15–30 g) were required to elevate GLP-1 levels, potentially because the majority of glutamine would be degraded or absorbed before reaching the distal gut. To deliver a higher proportion of glutamine to the distal gut, we tested the effect of glutamine capsules with an enteric coat for ileal release. Ten 600 mg capsules (6 g total) were administered to healthy or type 2 diabetic participants in the fasting state or together with an oral glucose tolerance test. An elevated peak GLP-1 level was measured at 90 min in the fasting state, but this did not translate into improved glucose tolerance or increased GLP-1 levels after the glucose drink [61]. As this was already a high capsule load for the participants, we concluded that delivering glutamine to the ileum by capsules is unlikely to be a useful future treatment for diabetes or obesity. The lack of efficacy of 6 g glutamine capsules is consistent with a study employing duodenal infusions, which concluded that 15 g but not 7.5 g of glutamine, delivered over 30 min, had a small stimulatory effect on GLP-1 but little effect on plasma glucose [62].

Other amino acids have not been tested in capsular form, but duodenal infusion studies have been performed using leucine, tryptophan and monosodium glutamate. Duodenal leucine infusion (3.3 or 9.9 g over 90 min) did not increase GLP-1 or PYY in healthy humans [63]. Tryptophan infusion at rates of 1.6 or 3.3 g over 90 min in healthy control subjects triggered elevation of GLP-1 and PYY and reduced ad libitum food intake [64]. Monosodium glutamate (2 g) was investigated as part of a study on the effects of different tastants (also testing the bitter component quinine and the sweet chemical rebaudioside A). Tastants, either alone or in combination, were infused into the duodenum of healthy volunteers, but none had measured effects on plasma GLP-1, PYY or CCK concentrations [65]. Of the amino acids tested, the results therefore suggest that tryptophan, which was effective at a relatively low dose, might be the best candidate for future encapsulation studies.

### 6.3 Bitter substances

In the duodenal tastant infusion study described above, the bitter agent quinine was tested at a dose of 75 mg [65], but had no effect on gut hormone levels. In another study, bitter secoiridoids from the *Gentiana lutea* root were tested in a microencapsulated formulation mixed into a vanilla pudding, and administered to healthy subjects. Concentrations of gut hormones, including GLP-1, GIP and PYY, were not significantly different after eating pudding containing microencapsulated bitter ingredients compared with control pudding, although subsequent energy intake was reduced [66]. Taken together with the duodenal infusion studies, however, the results do not suggest that bitter substances will provide a strong encapsulatable stimulus for targeting GLP-1 and PYY secretion.

### 6.4 Mono-oleoylglyerol and bile acids

Using the same study design as that employed to test glutamine capsules [61], we investigated the effects of encapsulated 1-mono-oleoyl glycerol (MOG), and the bile acid sodium taurocholate (STC). MOG was selected as a known ligand of the enteroendocrine cell receptor GPR119 that has been linked to GLP-1 secretion in a variety of in vitro and in vivo studies [67], [68]. MOG is a component of olive oil and a digestion product of triglycerides, and was generally regarded as safe for use in humans. Sodium taurocholate was selected as a ligand for the bile acid receptor GPBAR1 that is also strongly linked to GLP-1 secretion in many studies [5], [69]. Whilst some other bile acids such as sodium lithocholate are more potent GPBAR1 ligands, sodium taurocholate has previously been given safely to human subjects in oral and rectal forms [40], [70].

In brief, consented healthy volunteers were recruited to take a single dose of encapsulated stimulus or placebo followed by 4 h of intermittent blood sampling to identify changes in GLP-1 secretion, using the same protocols as those described for glutamine capsules [61]. The study took place at the Wellcome Trust Clinical Research Facility at Addenbrooke’s Hospital, Cambridge and was given ethical approval (Reference 12/EE/0389; 25/09/2012). The development and manufacture of the capsules was performed by Encap Drug Delivery Ltd (Livingston, UK), with capsules containing either 560 mg of MOG or 375 mg of STC or 300 mg of microcrystalline cellulose (placebo). The capsules were manufactured with an enteric coating designed to promote capsule release approximately 20 min after exposure to an alkaline environment. Initial participants received increasing numbers of active capsules on each visit, separated by a wash-out period of at least 1 week. Only results for the maximum dose (10 active capsules) are presented. Hunger, satiety and fullness were associated using a visual analogue scale (VAS).

**Fig. 1:**
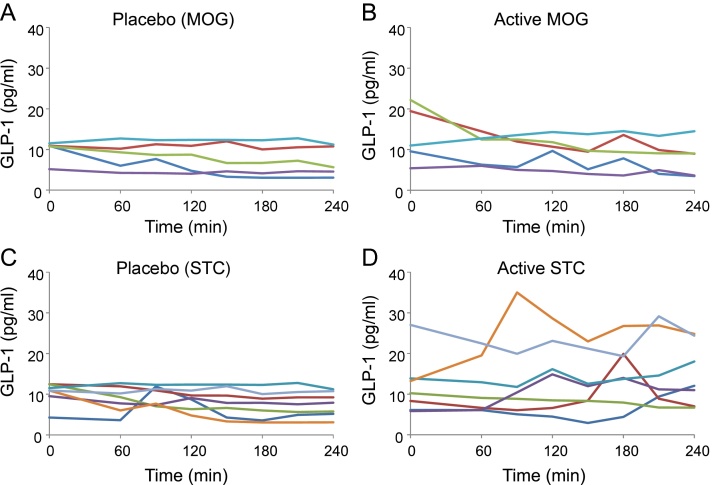
Effects of Monoacylglycerol (MOG) and sodium taurocholate (STC) capsules on GLP-1 levels in healthy humans. A,B. The effect of 10 capsules of either placebo (A) or MOG (5.6 g, B), delivered at time = 0, on plasma concentrations of GLP-1. Data from individual participants are shown: the same participant is shown by the same colour in A and B (n = 5). C,D. The effect of 10 capsules of either placebo (C) or STC (3.75 g, D), delivered at time = 0, on plasma concentrations of GLP-1. Data from individual participants are shown: the same participant is shown by the same colour in C and D (n = 7).

Five participants received the maximum dose of 10 capsules (5.6 g) of MOG. There was no evidence to support an increase in GLP-1 secretion (Fig. 1) or altered hunger, fullness or satiety following capsule ingestion. Although we had originally planned to recruit more participants, the study had to be terminated early due to technical problems with capsule production. MOG had a tendency to leak from capsules during manufacture, preventing satisfactory adherence of the band that normally encircles the joint between the two capsule shells. In vitro testing showed that even normal-looking capsules did not perform well on stability testing and disintegrated too early, and suggested that at least 10–30% of capsules would rupture in an acid environment comparable to the stomach, and would not therefore reach their target site in the ileum. The trial was terminated because results from the first 5 patients showed no hint of a GLP-1 response, and because we could not be certain whether the capsules were actually delivering MOG to the ileum as planned. The results did not, however, look promising.

Seven participants completed a dose ranging study for sodium taurocholate, and their individual responses to 10 capsules (3.75 g STC) ingested in the fasting state are shown in Fig. 1. Some participants showed little change in GLP-1 over the course of the test, but 4/7 exhibited a peak GLP-1 level that was >50% above baseline (mean 1.8-fold peak above baseline, p = 0.04, n = 7). No differences between active and placebo capsules were observed for hunger, fullness or nausea. Unfortunately, the STC capsules also performed poorly on initial stability testing and were only available for clinical testing for two months prior to shelf life expiration, making it unfeasible to study effects of the STC capsules in greater depth. Later assessment indicated that stability under standard conditions (25 °C/60% humidity) was acceptable, supporting a shelf life of 78 weeks. The results suggest that ileal capsular delivery of sodium taurocholate might have beneficial effects on gut hormone levels that warrant further testing.

## 7 Conclusions

There is relatively strong evidence in support of the idea that increasing the delivery of nutrients or bile acids to the distal intestine would stimulate GLP-1 and PYY secretion, and the clinical results of bariatric surgery suggest that this would in turn have beneficial effects on body weight, satiety and glucose metabolism. Most attempts to stimulate GLP-1or PYY release by delivering candidate stimuli in capsular or pelleted forms that shield them against proximal digestion and absorption have only, however, had limited success. We believe this is most likely because most nutrient sensing receptors have relatively low affinity for natural nutrient ligands that are normally present in the gut at high concentrations after a meal. To deliver stimulatory levels of these chemicals to the distal gut would require a correspondingly large capsular volume, and, for nutrients, consequent delivery of a significant caloric load. Bulk delivery of nutrients to the distal intestine is also associated with undesirable side effects such as bloating that might reduce patient compliance. Future attempts might therefore wish to focus on the identification of higher affinity ligands for these nutrient receptors, or the targeting of receptors that normally detect substances present at only low concentrations. Of the chemical entities described in this review, lauric acid, bile acids and tryptophan appeared the most promising, and potentially deserving of further study.
